# Blinding in Clinical Trials: Seeing the Big Picture

**DOI:** 10.3390/medicina57070647

**Published:** 2021-06-24

**Authors:** Thomas F. Monaghan, Christina W. Agudelo, Syed N. Rahman, Alan J. Wein, Jason M. Lazar, Karel Everaert, Roger R. Dmochowski

**Affiliations:** 1 Department of Urology, University of Texas Southwestern Medical Center, Dallas, TX 75390, USA; 2 Division of Cardiovascular Medicine, Department of Medicine, SUNY Downstate Health Sciences University, Brooklyn, NY 11203, USA; christina.agudelo@downstate.edu (C.W.A.); Jason.lazar@downstate.edu (J.M.L.); 3 Department of Urology, Yale University School of Medicine, New Haven, CT 06520, USA; syed.rahman@downstate.edu; 4 Division of Urology, Perelman School of Medicine at the University of Pennsylvania, Philadelphia, PA 19104, USA; alan.wein@pennmedicine.upenn.edu; 5 Department of Human Structure and Repair, Ghent University, 9000 Ghent, Belgium; karel.everaert@uzgent.be; 6 Department of Urological Surgery, Vanderbilt University Medical Center, Nashville, TN 37232, USA; roger.dmochowski@vumc.org

**Keywords:** bias, blinding, clinical trials, double, single, triple

## Abstract

Blinding mitigates several sources of bias which, if left unchecked, can quantitively affect study outcomes. Blinding remains under-utilized, particularly in non-pharmaceutical clinical trials, but is often highly feasible through simple measures. Although blinding is generally viewed as an effective method by which to eliminate bias, blinding does also pose some inherent limitations, and it behooves clinicians and researchers to be aware of such caveats. This article will review general principles for blinding in clinical trials, including examples of useful blinding techniques for both pharmaceutical and non-pharmaceutical trials, while also highlighting the limitations and potential consequences of blinding. Appropriate reporting on blinding in trial protocols and manuscripts, as well as future directions for blinding research, will also be discussed.

## 1. Introduction

Randomized clinical trials are a gold standard in evidence-based medicine because findings from these studies reflect the highest possible level of evidence which may be garnered from an original research study [1]. Randomized clinical trials tend to be highly tailored to a specific research question but, for a vast majority of interventions and outcomes, blinding is widely viewed as a core tenet of sound clinical trial study design [2,3,4]. 

Despite exponential growth in the number of clinical trials conducted yearly over the past two decades [5], multiple authors contend that the methodological quality of clinical trials has remained stagnant or even declined [6,7], such that true practice-guiding evidence on a broad range of medial topics paradoxically lags behind [8,9,10]. Blinding is one aspect of clinical trial design that remains particularly underutilized—although this methodological feature is not universally attainable, blinding is still implemented in only a fraction of clinical trials in which it is, in fact, deemed feasible [11,12,13]. Accordingly, it stands to reason that greater emphasis on addressing pervasive misconceptions about blinding in medical research is key to reconciling the growing divide between current research trends and actual practice needs [14]. Furthermore, because blinding is relevant to data analysis in the broadest sense, a sound understanding of blinding should be considered a prerequisite for evidence-based best practice, and thus of equal importance to providers and patients alike [15,16].

This article will review general principles for blinding in clinical trials, including examples of useful blinding techniques for both pharmaceutical and non-pharmaceutical trials, while also highlighting the limitations and potential consequences of blinding. Appropriate reporting on blinding in trial protocols and manuscripts, as well as future directions for blinding research, will also be discussed. Note that this article will focus on blinding in clinical trials, where it is most often discussed, but the relevance of blinding spans the gamut of study designs, from late-stage randomized interventional trials to retrospective observational studies (e.g., blinded outcome assessors) [17,18].

## 2. What Is Blinding?

In an unblinded, or “open”, study, information about the assigned interventions is available to all people and groups involved in the research. Blinding, or “masking”, is the process by which information that has the potential to influence study results is withheld from one or more parties involved in a research study. 

Importantly, the topic of blinding must be distinguished from allocation concealment. Allocation concealment is the process by which investigators and participants enrolled in a clinical study are kept unaware of upcoming group assignments until the moment of assignment [19]. Allocation concealment is a core tenet of proper study randomization and plays a key role in preventing selection bias [20]. Blinding, in contrast, refers to the act of withholding information about the assigned interventions from people involved in the trial from the time of group assignment until the experiment is complete. While proper randomization minimizes the differences between treatment groups at the beginning of a trial, it does not prevent differential treatment of study groups during the trial, nor does it prevent differential interpretation and analysis of study outcomes [21].

## 3. Why Do We Blind?

We blind because the potential for bias is everywhere. Bias can take numerous shapes and forms when people involved in a research study are privy to information about the assigned interventions [22]. Participant knowledge of their group allocation can bias expectations, adherence to the trial protocol, treatment-seeking behavior outside the trial, and assessment of the effectiveness of an intervention [23]. Differential treatment, attention, or attitudes toward subjects by a non-blinded healthcare team or other members of the research staff also pose a major threat to unbiased outcomes [24,25]. Importantly, once bias is introduced from any one of these potential sources, there exist no analytical techniques by which to reliably correct for this limitation [21].

Several lines of empirical evidence demonstrate the direct effects of non-blinding on clinical trial outcomes. One systematic review from Hróbjartsson et al. concluded that attrition is significantly more frequent among controls versus subjects assigned to the experimental group when participants are not blinded—a phenomenon not common to well-designed participant-blinded trials [26,27]. Moreover, participant-reported outcomes were found to be exaggerated by 0.56 standard deviations overall in trials of non-blinded versus blinded participants, with an even greater discrepancy in trials investigating invasive procedures [26]. In three separate meta-analyses from Hróbjartsson et al. on observer bias in randomized clinical trials, non-blinded versus blinded outcome assessors were found to generate exaggerated hazard ratios by an average of 27% in studies with time-to-event outcomes [28], exaggerated odds ratios by an average of 36% in studies with binary outcomes [29], and a 68% exaggerated pooled effect size in studies with measurement scale outcomes [30]. Taken together, the four meta-analyses from Hróbjartsson et al. indicate that participant blinding and assessor blinding similarly lend to exaggerated effect sizes, although the three analyses on observer bias collectively suggest that the type of variable assessed influences how large of an effect blinding may have on study results.

The relevance of blinding in mitigating bias is perhaps most easily appreciated in studies involving subjective outcomes. However, many seemingly objective outcomes rely on interpretation of participant data and thus are also characterized by subjective elements (e.g., electrocardiogram scan interpretation for myocardial infarction) [31]. Further, even unequivocally objective outcomes, such as time to death, can be indirectly affected by factors such as the use of advance directives, concurrent interventions, and follow-up intensity [31]. Correspondingly, while some meta-analyses have reported more robust evidence of bias with subjective versus objective outcomes [32], this finding is inconsistent, and multiple other studies have reported no appreciable difference in estimated treatment effect based on the degree of outcome subjectivity [29,33]. Thus, for both subjective and objective outcomes, current evidence suggests that blinding can play a potentially major role in mitigating threats to internal and construct validity [34].

## 4. Who and What Do We Blind?

Current literature has identified as many as 11 distinct groups meriting unique consideration when it comes to blinding: (1) participants, (2) care providers, (3) data collectors and data managers, (4) trial managers, (5) pharmacists, (6) laboratory technicians, (7) outcome assessors (study personnel who collect outcome data), (8) outcome adjudicators (personnel who confirm that outcomes meet prespecified criteria), (9) statisticians, (10) members of safety and data monitoring committees, and (11) manuscript writers [35]. 

In a blinded clinical study, treatment assignment is the information most frequently withheld from these groups [35]. However, in many cases, blinding of some of the aforementioned groups to additional information is also feasible. For example, laboratory technicians, outcome assessors, and outcome adjudicators may also be blinded to basic demographic and clinical characteristics of the study population, as well as the overall purpose of the trial [35]. 

Consistent with the significant heterogeneity as to “who” and “what” may be blinded, it is important to appreciate blinding on a graded continuum rather than as an all-or-nothing phenomenon, wherein the blinding of some study groups to pertinent information as feasible (i.e., “partial blinding”) can tangibly improve the strength of trial results—even when maximal blinding of all study groups cannot be achieved [35,36]. 

## 5. How Do We Blind?

A multitude of techniques have been described for blinding all people and groups involved in clinical trials. The researcher’s specific approach to blinding will ultimately be highly dependent on the specific parties being blinded as well as the research question and intervention at hand. In fact, there exists considerable flexibility in blinding—even beyond the strategies for blinding subsequently highlighted in this section, investigators may feasibly create their own novel blinding technique, so long as (1) the technique successfully conceals pertinent information about the groups and (2) does not impair the ability to accurately assess or adjudicate outcomes [37].

Boutron et al. systematically reviewed blinding in randomized control trials assessing pharmacologic treatments, organizing their results to provide an excellent inventory of practical methods to (1) establish blinding of participants and providers, (2) maintain blinding (i.e., prevent unblinding), and (3) blind outcome assessors [38]. Common methods to establish participant/provider blinding include centralized preparation of similar capsules or tablets, bottles, and syringes; flavoring to mask the specific taste of active oral treatments; and double-dummy procedures. (A double-dummy technique is the use of more than one placebo for the maintenance of blinding, particularly in cases when two treatments under investigation cannot be made identical, wherein subjects are assigned to different sets of treatment and more than one group may receive placebo. For example, in a trial designed to compare an oral tablet medication with a medication administered by intramuscular injection, an indistinguishable placebo can be prepared for both the tablet and injection, and one group may receive the active medication tablet and placebo injection, with another group receiving the placebo tablet and active medication injection.) Strategies for reducing the risk of unblinding include centralized dosage adaptation as warranted, centralized evaluation for side effects, partial information about side effects, and use of an “active placebo” (sugar pill which mimics expected side effects of the active treatment). Methods for blinding outcome assessors typically rely on centralized assessment of complementary investigations, clinical examinations, and adjudication of clinical events.

**Figure 1:**
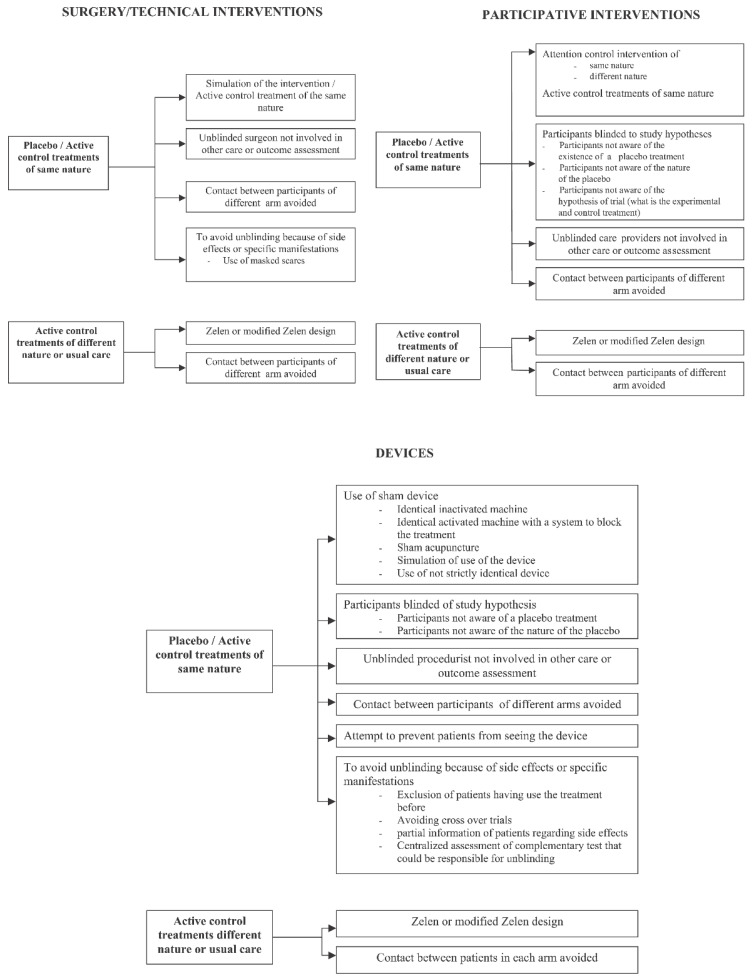
Sham procedure performed according to the category of treatment assessed. Reprinted with permission from ref. [43]. Copyright 2007 Boutron et al. Full text available from https://journals.plos.org/plosmedicine/article?id=10.1371/journal.pmed.0040061#s5.

Blinding in non-pharmaceutical trials is undoubtedly faced with several unique challenges related to the complexity and physical component of such interventions, participant and physician acceptance, and broader ethical and safety considerations [39,40]. Accordingly, relative to pharmaceutical trials, blinding is typically implemented even less frequently in those investigating surgical procedures, medical devices, and participative interventions (e.g., rehabilitation) [41]. Nevertheless, compared to pharmaceutical trials, blinding in these trials is of no less relevance in the pursuit of true practice-shaping evidence [41,42]. In fact, blinded interventional trials are often practical, and may even feasibly involve a placebo group (i.e., “sham procedure”) [13]. Figure 1 provides examples of sham procedures for surgical interventions and other non-pharmacological clinical trials, as published in a separate systematic review from Boutron and colleagues [43].

In trials comparing two similar invasive procedures, particularly those performed under general anesthesia or heavy sedation, blinding of participants can be relatively straightforward [21]. Notably, however, blinding of participants may even be feasible when surgical interventions differ significantly. Namely, there exist several well-described recent examples of investigators devising highly creative solutions to maintain participant blinding in invasive interventional trials, including imitation of the surgical access point, replication of visual, auditory, and physical cues in the operating room, matching the duration of experimental and control procedures, and standardization of additional care (e.g., diagnostic scans, perioperative medical management, etc.) [13]. 

Beyond these measures, a handful of studies have even managed to blind surgeons to the intervention being performed. For example, in one randomized control trial of electrothermal therapy for chronic lower back pain, surgeons inserted an intradiscal catheter under fluoroscopic guidance in all cases, at which point an independent technician connected the catheter to a generator and delivered either electrothermal energy (experimental group) or did not (control group) [44]. A trial on palatal implants for obstructive sleep apnea blinded proceduralists through the use of a manufacturer-preloaded delivery system containing either an implant (active treatment) or no implant (sham) [45]. While blinding of surgeons is seldom practical, eliminating their role in post-operative care, follow-up, and additional treatment is often feasible for minimizing this potential source of bias [46]. 

**Figure 2:**
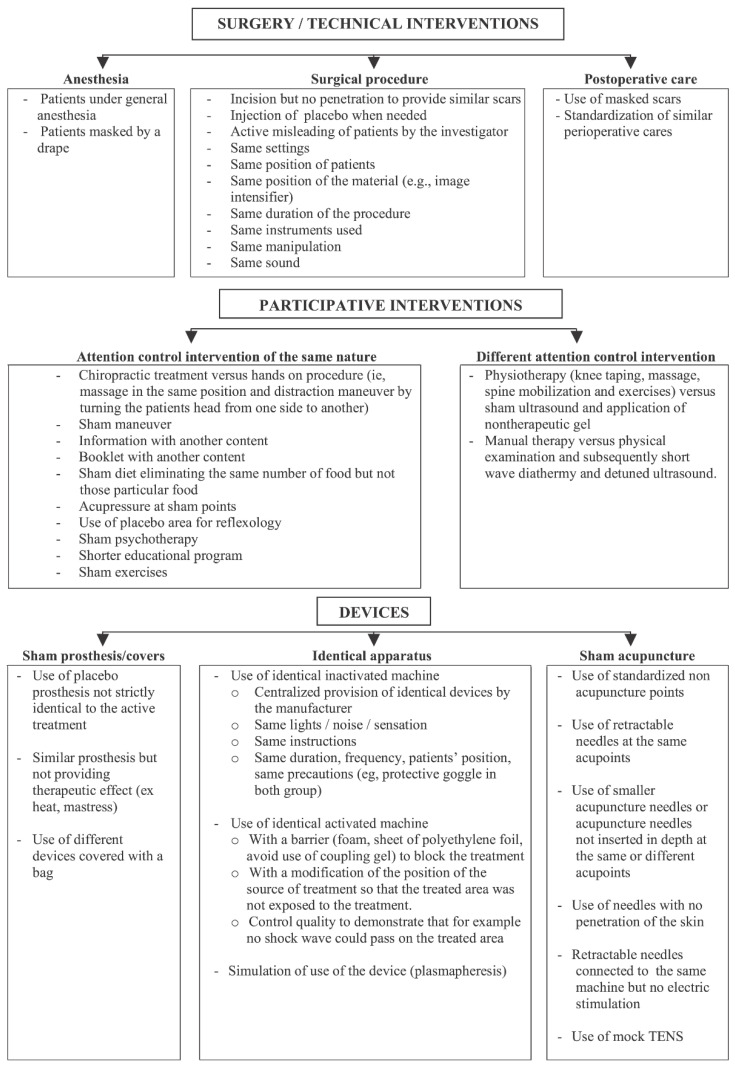
Methods of blinding participants, health care providers, or other caregivers that rely on the category of treatment and comparator assessed. Reprinted with permission from ref. [43]. Copyright 2007 Boutron et al. Full text available from https://journals.plos.org/plosmedicine/article?id=10.1371/journal.pmed.0040061#s5.

Even in the absence of surgeon blinding, it is often possible to blind other members of the care team and study staff from information that has the potential to bias study results. Simple measures such as uniform dressings large enough to cover all potential incision sites have been used to successfully blind other members of the post-operative care team [21]. Blinding of outcome assessors, while uncommonly performed in surgical trials, is frequently practical through simple techniques such as the use of independent assessors, concealed incisions, and blinding of digital images [12]. Figure 2 depicts methods for blinding key groups in randomized control trials for different non-pharmacological clinical trials from Boutron et al. [43].

## 6. Limitations of Blinding

Although blinding is generally viewed as a very effective method by which to eliminate bias, blinding does pose some inherent limitations, and it behooves clinicians and researchers to be aware of such caveats. Blinding often requires considerable effort and expense [47]. Blinding also has a well-established negative impact on study recruitment [48,49,50]. Additionally, blinding inherently deviates from real-world experience, making it a hallmark feature of trials which aim to maximize the likelihood of establishing the efficacy of an intervention by testing it in an ideal setting (i.e., “explanatory trials”), but potentially less relevant for trials which aim to generate situations that are as close to routine practice as possible (i.e., “pragmatic trials”) [51]. Blinding has also been suggested to potentially adversely impact subsequent care after the conclusion of a clinical trial [52].

In contrast to open-label studies, blinded clinical trials are inherently susceptible to the phenomenon of unblinding (i.e., “code-breaking”). The term “unblinding” is often used to describe the formal process by which subjects and/or investigators are made aware of a participant’s treatment assignment according to prespecified contingencies (e.g., in the case of a medical emergency which compromises a participant’s safety). However, even in the absence of formal unblinding, subjects in either the intervention or control group may feasibly come to suspect their assignment status using more subtle clues, such as the presence (or absence) of signature medication effects or side effects [53,54]. In fact, potential threats to blinding are pervasive and multifaceted—there have been documented cases of researchers intentionally subverting blinding by comparing pills or viewing restricted notes and, more recently, instances of trial participants connecting through social media and collaborating to deduce their treatment allocation [55,56,57]. 

Unblinding has several deleterious effects that can threaten the validity of trial results [58,59]. Subjects in a placebo arm who discover or suspect that they are not receiving active treatment may become upset or uncooperative (i.e., “resentful demoralization”), access interventions outside of the trial (i.e., “compensatory rivalry”, which inherently increases the risk of “contamination” (i.e., when members of the control group are inadvertently exposed to the intervention)), exaggerate negative responses (i.e., “biased event reporting”), or even withdraw from the trial [60,61]. Similarly, empathetic caregivers who know subjects to be controls may provide them with non-study, but effective, interventions (i.e., “cointerventions”) [62]. Conversely, participants who suspect or know that they are on the “better” treatment may downplay mild side-effects, while clinicians with this information may downplay participants’ symptoms and underreport “soft” clinical findings (also “biased event reporting”, but typically in the opposite direction compared to controls) [62]. 

Multiple recent analyses of blinded trials published in high-impact medical journals have concluded that the implementation of blinding is inconsistent and successful in perhaps fewer than 50 percent of cases [62]. Various efforts have been made to quantitively assess blinding, which most commonly utilize a blinding questionnaire or survey, and ask subjects in both the experimental and control groups to guess their treatment allocation [63]. Several methods have been employed in analyzing these data, including chi-square and McNemar’s tests, a standard Kappa statistic, and multiple blinding indices [63,64,65]. However, it should be noted that an assessment of blinding success is only seldom performed [66,67,68], and has even been criticized for the inherent limitations of this process [69]—many of which centering on the fact that end-of-trial tests for “blindness” cannot be reliably distinguished from hunches about efficacy [62]. In other words, participant responses to end-of-trial blinding surveys are likely influenced by prior assumptions and expectations regarding treatment efficacy, such that beliefs about allocation may still cause bias even when blinding succeeds in making these beliefs independent of actual allocation [70]. Citing these reasons, the most recent “Current Consolidated Standards of Reporting Trials” (CONSORT) statement no longer advocates for testing of blinding success, reflecting a major divergence from prior renditions of CONSORT guidelines [71]. The topic of evaluating and reporting blinding success remains debated and is highly complex, but there does exist a relative consensus regarding the need for a greater understanding of the bias-generating consequences that result from its loss, irrespective of whether they arise from the loss of blindness, per se, or rather from beliefs about allocation or another cause [62].

The limitations of blinding with respect to recruitment, applicability to routine practice, and analysis have led some authors to challenge the role of participant and clinician blinding as a universal gold standard in evidence acquisition. Anand et al. emphasize that blinding of participants and clinicians requires careful consideration of the negative effects of blinding against its potential benefits, as guided by the following key questions: (1) whether blinding is needed for a scientifically sound result; (2) whether changes in participant or clinician awareness of assignment status will cause a change in behavior that influences results; (3) whether there is a risk of excessive harm with blinding and, if so, whether said risk is justified by the importance of the study findings; and (4) whether the financial cost of blinding compromises spending on other aspects of trial integrity [53]. Note that recent criticisms of blinding from Anand et al. and others primarily center on the topic of participant and/or clinician blinding—there remains a relative consensus regarding the critical importance of objective outcomes, blinded outcomes assessment, and blinded adjudication of outcomes in mitigating major sources of bias in clinical trials [53]. 

## 7. Blinding: Reporting Responsibly

The terms single-blind, double-blind, and triple-blind are often used to describe studies in which one, two, or three parties, respectively, are blinded to information about the treatment groups. Recall, however, that up to 11 discrete groups merit unique consideration with respect to blinding in clinical trials [35]. Correspondingly, there has long existed great variability in textbook definitions and clinician interpretations of these terms [72], which is particularly problematic given that study authors often fail to specify who, exactly, has been blinded [73]. For example, a sample of randomized clinical trials published in 2001 found that more than half of “double-blind” studies failed to describe the blinding status of any person involved in the trial [74]. Moreover, on a follow-up survey sent to trial authors, 15 different operational meanings of the term “double-blind” were reported by the investigators, who typically believed that their preferred definition was the most widely used [74]. 

In view of the high potential for misinterpretation, authors of the most recent CONSORT (2010) statement instruct researchers to “abandon [the] use” of “double-blind” and related terms [71]. Instead, the 2010 CONSORT guidelines direct authors to “explicitly report blinding status”, including who is and is not blinded, what information is concealed, and how blinding is performed [75]. Further, if relevant, authors must provide a description of the similarity of the interventions and procedures used for blinding [75]. (Specification of how blinding was performed, as well as a description of an intervention’s similarity, were both “noteworthy specific changes” from early renditions of the CONSORT statement [75], motivated by the need for greater “evidence of the method of blinding” [71].) The “Standard Protocol Items: Recommendations for Interventional Trials” (SPIRIT) 2013 statement similarly directs authors to specify who will be blinded and how blinding will be accomplished in clinical trial protocols [76]. 

Despite these increasingly explicit consensus recommendations, there still exist major discrepancies in how blinding is reported in registered protocols and publications, as evidenced by continued widespread suboptimal adherence to current CONSORT and SPIRIT guidelines [77,78,79]. Several strategies have been proposed for improving the quality of reporting on blinding in clinical trials. One practical option recently proposed by Lang et al. is to detail blinding status using a standardized “Who Knew” table [35]. Although such a practice has not yet gained widespread traction, the author’s table aptly illustrates the extent to which blinding should be described to ensure transparency in research methodology (Table 1).

## 8. Future Directions

Numerous studies have used and not used blinding. Comparatively, however, far fewer papers have attempted to comprehensively review blinding in clinical trials, and several questions remain unanswered. The magnitude of the estimated treatment effect associated with participant blinding status has been shown to vary considerably across different studies [29]. As detailed previously, the three separate meta-analyses from Hróbjartsson et al. on observer bias collectively suggest that the type of variable also influences the magnitude of the effect which blinding may exert on study results [28,29,30]. Further, a subset of studies have found non-blinded assessors to significantly favor control, rather than experimental, interventions, corresponding to a comparable degree of observer bias in the opposite direction, but the reason for this remains unclear [30]. Moreover, compared to participants and outcome assessors, the impact of blinding of other trial personnel and healthcare professionals on estimated treatment effect is even less well-established [32,33]. Therefore, multiple factors appear to impact the magnitude of bias imposed by a lack of blinding, and recent meta-epidemiological evidence suggests that many relevant study factors remain incompletely characterized in this regard [33]. The effects of unblinding all above-mentioned study groups on study outcomes likewise remain poorly characterized. 

There exist several additional facets of clinical trial study design which also merit greater investigation in relation to blinding status. Historically, placebos constituted the primary comparator arm in most pharmacologic randomized control trials, but trials involving active best-of-care comparator arms and other non-placebo background therapies have grown in popularity in recent years [81,82]. Surgical trials are seemingly even more heterogeneous in this regard, as new surgical interventions may be tested against placebo (i.e., “sham procedure”), but also against a similar surgical/invasive intervention, dissimilar surgical/invasive intervention, pharmacotherapy, participative intervention (e.g., physical therapy), or active surveillance/watchful waiting [41]. Accordingly, whether specific characteristics of a study’s comparator arm(s) modify the effects of blinding or consequences of unblinding merits further study [83]. Additionally, although blinding is infrequently incorporated into early-stage clinical trials [84], we are unaware of studies assessing the effects of blinding as a function of study phase, and it may be revealing to assess the relative effect of blinding in phase 2 versus phase 3 trials—particularly in cases where phase 2 and phase 3 trials show divergent results [85]. We also advocate for a more simplified and standardized approach to incorporating blinding in power analyses and sample size re-estimation for adaptive trials [86,87,88].

## Figures and Tables

**Table 1:** A standard table for reporting the use of blinding in randomized trials of pharmaceutical interventions.

Group or Individual Blinded ^a^	Information Withheld ^b^	Method of Blinding ^c,d^	Blinding Compromised
Required fields to be completed for all trials described as blinded			
Person assigning participants to groups	Group assignment	Concealed allocation schedule	No
Participants	Group assignment	Placebo medications; sham surgeries	No
Care providers	Group assignment	Not told of group assignment	No
Data collectors and managers	Group assignment	Not told of group assignment	No
Outcome assessors	Purpose of study; group assignment; participant characteristics	Participants given numerical identifiers	No
Statisticians	Participant and group identities	Participants and groups given numerical identifiers	No
Supplemental fields for all blinded groups or individuals not mentioned above			
Trial manager	Not applicable	...	...
Pharmacists	Not applicable	...	...
Laboratory technicians	Participant identities	Participants given numerical identifiers	
Outcome adjudicators	Group assignment	Groups given numerical identifiers	Yes (put details in text)
Data monitoring and safety committees	Not applicable	...	...
Manuscript writers	Not blinded	...	...

(^a^) Other groups or individuals in a trial that were capable of being blinded should be listed in the table, and whether or not they were blinded in the study should be indicated. Individuals with dual responsibilities, such as caregiving and data collecting, should be identified by combining the entries in the same row heading. (^b^) Although group assignment is the information most commonly withheld in a blinded trial, data assessors, such as pathologists and radiologists, are often blinded to the purpose of the trial, group assignment, and the demographic and clinical characteristics of participants whose biopsy samples or images they are interpreting. (^c^) In many cases, authors should determine before the trial begins whether the method of blinding had a reasonable chance of being effective, including establishing the similarity between active and placebo preparations and the bioequivalent availability for two or more active drugs [80]. Testing the effectiveness of blinding after the trial has ended is uninformative because the results cannot be separated from pre-trial expectations of the success of the intervention [47]. (^d^) If blinding has been compromised, authors should report the fact and indicate the potential implications the loss of blinding might have for interpreting the results [80]. Reprinted with permission from ref. [35]. Copyright 2020 Lang et al. Full text available from https://trialsjournal.biomedcentral.com/articles/10.1186/s13063-020-04607-5.

## Data Availability

Not applicable.
